# Exploring the Causal Relationship Between Antidepressant Use and Lung Cancer Risk: A Mendelian Randomization Analysis

**DOI:** 10.1111/crj.70102

**Published:** 2025-07-02

**Authors:** Chunli Yang, Wenlin Xu

**Affiliations:** ^1^ Department of Central Laboratory The Fourth Affiliated Hospital of Jiangsu University Zhenjiang Jiangsu China

**Keywords:** antidepressant, causal inference, genome‐wide association study, lung cancer risk, Mendelian randomization

## Abstract

**Purpose:**

The dramatic increase in antidepressant prescribing over the past decade has sparked debate about the possible contribution of antidepressants to elevated cancer risk. In this study, we investigate whether antidepressant use has a causal relationship with lung cancer risk.

**Methods:**

Genome‐wide association study (GWAS) data for antidepressant use were acquired from the FinnGen Biobank, while GWAS data for overall lung cancer and specific histological subtypes were obtained from the UK Biobank (UKBB) and IEU databases. The causal impact was evaluated using inverse variance weighting (IVW), MR‐Egger regression, and weighted median (WM) approaches. Multiple sensitivity analyses were conducted to validate the findings. Results are expressed as ORs and 95% CIs.

**Results:**

No causal relationship between antidepressant use and lung cancer risk was observed in the IVW (OR = 1.001, 95% CI = 0.999, *p* = 0.279), MR‐Egger (OR = 1.002, 95% CI = 0.992, *p* = 0.700), and WM analyses (OR = 1.000, 95% CI: 0.997, *p* = 0.889). Similar results were found across lung cancer subtypes, including lung adenocarcinoma (LUAD) (OR = 1.197, 95% CI = 0.884–1.619, *p* = 0.247), lung squamous cell carcinoma (LUSC) (OR = 1.052, 95% CI = 0.822, *p* = 0.688), and small cell lung carcinoma (SCLC) (OR = 1.874, 95% CI = 0.737, *p* = 0.187). Sensitivity tests confirmed the robustness of these results.

**Conclusions:**

This analysis indicates antidepressant use is not significantly associated with lung cancer risk.

## Graphical abstract

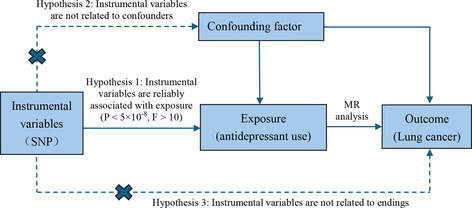

## 1 Introduction

Cancer remains a significant global health challenge. Based on the most recent data from the International Agency for Research on Cancer (IARC), lung cancer was the most prevalent cancer globally in 2022, with an estimated 2.5 million new cases, representing 12.4% of all newly diagnosed cancers. As the leading cause of cancer mortality, lung cancer was responsible for an estimated 1.8 million deaths, which represents 18.7% of all cancer deaths globally [1]. In China, recent data from the National Cancer Center indicate that among all malignant tumors, lung cancer has the highest incidence and death rates, and its incidence rate continues to increase [2]. Lung cancer risk factors include smoking, chronic inflammation, occupational exposure, family history, and genetic predisposition; however, these factors are generally nonmodifiable. Therefore, identifying and addressing potentially modifiable risk factors is crucial for lung cancer prevention.

Among common pharmacological treatments for depression are selective serotonin reuptake inhibitors (SSRIs), tricyclic antidepressants (TCAs), and norepinephrine reuptake inhibitors (SNRIs). Over the past decade, antidepressant prescriptions have significantly increased worldwide, sparking debate over the possible associations between antidepressant use and cancer risks [3, 4, 5, 6, 7]. Rodent studies have produced mixed results regarding this association. L.J. Brandes and colleagues observed tumor‐promoting effects at clinically relevant doses of antidepressants such as amitriptyline and fluoxetine hydrochloride [8]. However, subsequent research by R.A. Bendele and colleagues found no increase in spontaneous tumor incidence among rats and mice treated with fluoxetine hydrochloride [9]. Clinical studies have similarly been inconsistent. Some research indicates a potential increased cancer risk associated with antidepressant use; for example, research using a population‐based case–control design reported a high breast cancer risk in patients treated with TCAs for over 2 years compared to non‐users (OR = 2.1, 95% CI = 0.9–5.0). The application of paroxetine was linked to a marked rise in breast cancer risk (OR = 7.2, 95% CI = 0.9–58.3) [10, 11, 12]. Conversely, other studies do not support this association, including a recent Mendelian randomization (MR) study on breast cancer, which reported no significant association between antidepressant or SSRI application and breast cancer risks, whether ER‐positive or ER‐negative [13, 14, 15, 16, 17, 18, 19].

Previous reports on the associations between antidepressants and lung cancer risk have also yielded mixed results [12, 20, 21, 22]. Observational studies are prone to potential biases, including reverse causality and confounding, which can distort the association between exposure and disease. Over the past few decades, genome‐wide association studies (GWAS) have become a vital tool in genetics research. MR is a statistical method that uses GWAS data to identify single‐nucleotide polymorphisms (SNPs) as instrumental variables (IVs) for exposures, such as hormone levels, resembling the process used in a randomized controlled trial (RCT) [23, 24, 25, 26, 27]. This approach allows for the investigation of causal relationships between exposures and outcomes while reducing the influence of confounding factors and reverse causation [23, 24, 25] Herein, we employed a two‐sample MR approach using GWAS data to examine the causal relationship between antidepressant use and lung cancer risk.

## 2 Methods

### 2.1 Research Design

**FIGURE 1:**
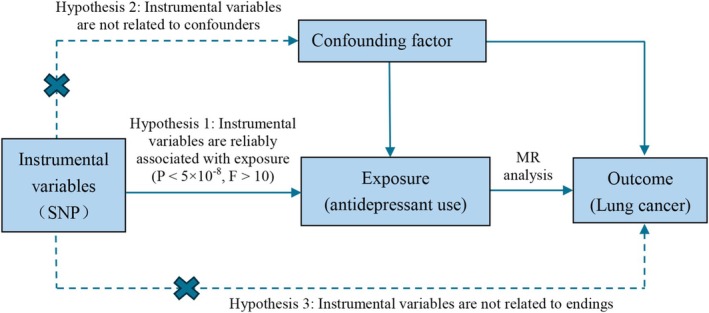
Schematic diagram of two‐sample Mendelian randomization analysis.

SNPs related to antidepressant use were extracted from relevant GWAS and employed as IVs in a two‐sample MR analysis. This approach aimed to examine the causal relationships between antidepressant use and lung cancer risk. To assess the robustness of the causal inferences from this analysis, sensitivity tests were subsequently performed. The validity of two‐sample MR analysis relies on the fulfillment of three key assumptions: (i) the IVs must be significantly related to the exposure (antidepressant use), (ii) the IVs should remain unaffected by potential confounders, and (iii) the IVs must be unrelated to the outcomes (lung cancer risk), except through the exposure. The relationships among these conditions are presented in Figure 1.

### 2.2 Data Resources

Study data were sourced from the GWAS database. Exposure data on antidepressant use were obtained from the FinnGen database, encompassing 235 818 samples and 21 299 063 SNPs. Outcome data for lung cancer were derived from the UK Biobank (UKBB), which included 372 487 samples and 11 078 115 SNPs. Given the heterogeneity across lung cancer pathology types, subtype‐specific data were also analyzed to assess the causal relationships between antidepressant use and different lung cancer subtypes in a subgroup analysis. GWAS summary data were retrieved from Wang et al. on the IEU OpenGWAS platform (IEU OpenGWAS project, mrcieu.ac.uk) [28], covering total lung cancer as well as specific subtypes: small cell lung cancer, squamous cell carcinoma, and adenocarcinoma. An overview of the GWAS data can be found in Table 1.

**TABLE 1:** Detailed information of the genome‐wide association data.

Phenotype	Population	GWAS ID	Sample size	Sources
**Exposure**				
Antidepressant	European	—	235 818	FinnGen
**Outcome**				
Lung cancer	European	ieu‐b‐4955	372 487	UK Biobank
Lung cancer	European	ieu‐a‐966	27 209	ILCCO
Adenocarcinoma	European	ieu‐a‐965	18 338	ILCCO
Squamous carcinoma	European	ieu‐a‐967	33 351	ILCCO
SCLC	European	finn‐b‐C3_SCLC	218 792	FinnGen

### 2.3 Identification of Genetic IVs

In this study, genetic IVs related to both exposure and outcome were selected from independent samples of individuals of the same race, meeting the key assumptions required for MR analysis. To ensure robust selection, we followed a rigorous process to identify the SNPs most strongly associated with the exposure variable: (1) SNPs were selected with a significant threshold of *p* < 5 × 10^−8^; (2) SNPs in linkage disequilibrium were excluded, using parameters of *r* ^2^ < 0.001 and a window size of 10 000 kb [29]; (3) SNPs with ambiguous or undefined directional effects (echo SNPs) were removed; (4) Only SNPs with a significant association with the exposure were retained, with an F‐statistic > 10 calculated as follows: *F* = *R* ^2^ (N‐2)/(1‐*R* ^2^), where N is the sample size of the GWAS for the exposure, *R* ^2^ represents the extent of phenotype variance influenced by the IV, calculated as: *R* ^2^ = 2 × (1‐EAF) × EAF × *β* ^2^, where EAF represents the frequency of the effect allele, while *β* denotes the SNP's effect size on the exposure [30].

### 2.4 MR Analysis

We employed three MR methods [Weighted Median (WM), MR‐Egger, and Inverse‐Variance Weighted (IVW)] to investigate potential causal relationships between antidepressant use and lung cancer. The primary MR analysis was performed using the IVW approach, which provides a comprehensive causal estimate between exposure and outcome, and demonstrates higher statistical efficiency when each genetic variant fulfills the IV assumptions [31]. MR‐Egger and WM analyses were used as complementary methods to test for causality and to check for consistency across methods [32]. *p* < 0.05 was deemed significant for two‐sided tests. All analyses were carried out using R software (v4.1.1) with the “TwoSampleMR” package (v0.6.6).

### 2.5 Sensitivity Analysis

To assure the robustness of our findings, multiple sensitivity tests were conducted. We assessed tool strength using the F‐statistic, which approximates the square of the SNP‐phenotype association divided by its variance. An F‐statistic of less than 10 indicates that the tool is likely to be weak. Power calculations were based on the approximation that the sample size of an MR study is the sample size of the outcome exposure divided by the *r* ^2^ of the genetic tool exposure, calculated using an online tool [33]. Cochran's Q‐test was used to examine heterogeneity, with *p* > 0.05 suggesting an absence of significant heterogeneity, supporting the validity of causal inferences [33]. MR‐PRESSO was utilized to determine and correct for directional pleiotropy by identifying and removing outlier SNPs [34]. In a large number of simulations, we evaluated the performance of MR‐PRESSO and compared it to other complementary methods, including methods that measure and correct for the average level of multidirectional effects across all variants, as well as outlier robust methods [33]. Horizontal pleiotropy was further evaluated using the MR‐Egger regression's intercept. Finally, a leave‐one‐out analysis was carried out to evaluate if any single SNP disproportionately affected the MR results [35].

## 3 Results

### 3.1 Instrumental Variables

Using the “TwoSampleMR” package, 37 SNPs were selected from the GWAS of a Finnish population that met the necessary inclusion criteria for IVs. Comprehensive details on these SNPs can be found in Table S1. The lowest F‐statistic among these IVs was 153, indicating that the selected genetic instruments are significantly associated with the exposure variable. This high F‐statistic suggests that the selected IVs are robust and minimize bias associated with weak instruments. Consequently, all 37 SNPs were included in the MR analysis, examining the relationships between antidepressant use and lung cancer risks across different histological subtypes.

### 3.2 Two‐Sample MR Analysis

**FIGURE 2:**
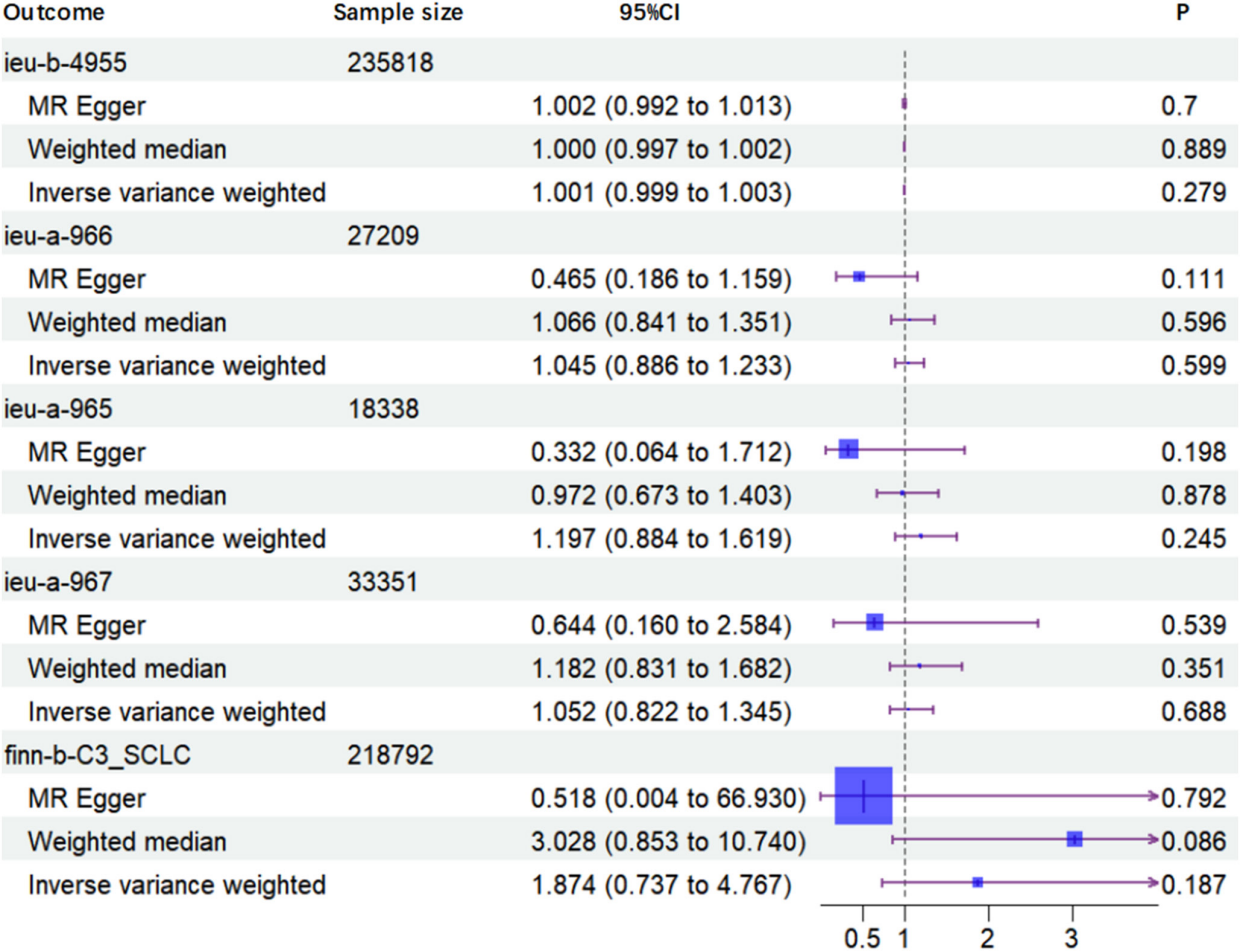
Results of Mendelian randomization analyses.

The primary analysis used the IVW method to determine the causal associations between antidepressant use and lung cancer risks. The IVW analysis indicated no significant association (OR = 1.001; 95% CI = 0.999–1.003; *p* = 0.279). Similar findings were observed with other MR methods, including the WM estimator (OR = 1.000; 95% CI = 0.997–1.002; *p* = 0.889) and MR‐Egger regression (OR = 1.002; 95% CI = 0.992–1.013; *p* = 0.700). These consistent findings across methods imply that antidepressant use is not causally associated with lung cancer risk. Furthermore, MR analysis revealed non‐significant causal relationships between antidepressant use and any lung cancer subtypes. Specifically, no significant associations were found for total lung cancer (OR = 1.045; 95% CI = 0.886–1.233; *p* = 0.599) or for the subtypes of lung adenocarcinoma (LUAD, OR = 1.197; 95% CI = 0.884–1.619; *p* = 0.247), lung squamous cell carcinoma (LUSC, OR = 1.052; 95% CI = 0.822–1.345; *p* = 0.688), and small cell lung carcinoma (SCLC, OR = 1.874; 95% CI = 0.737–4.767; *p* = 0.187) (Figure 2).

### 3.3 Sensitivity Analysis

**FIGURE 3:**
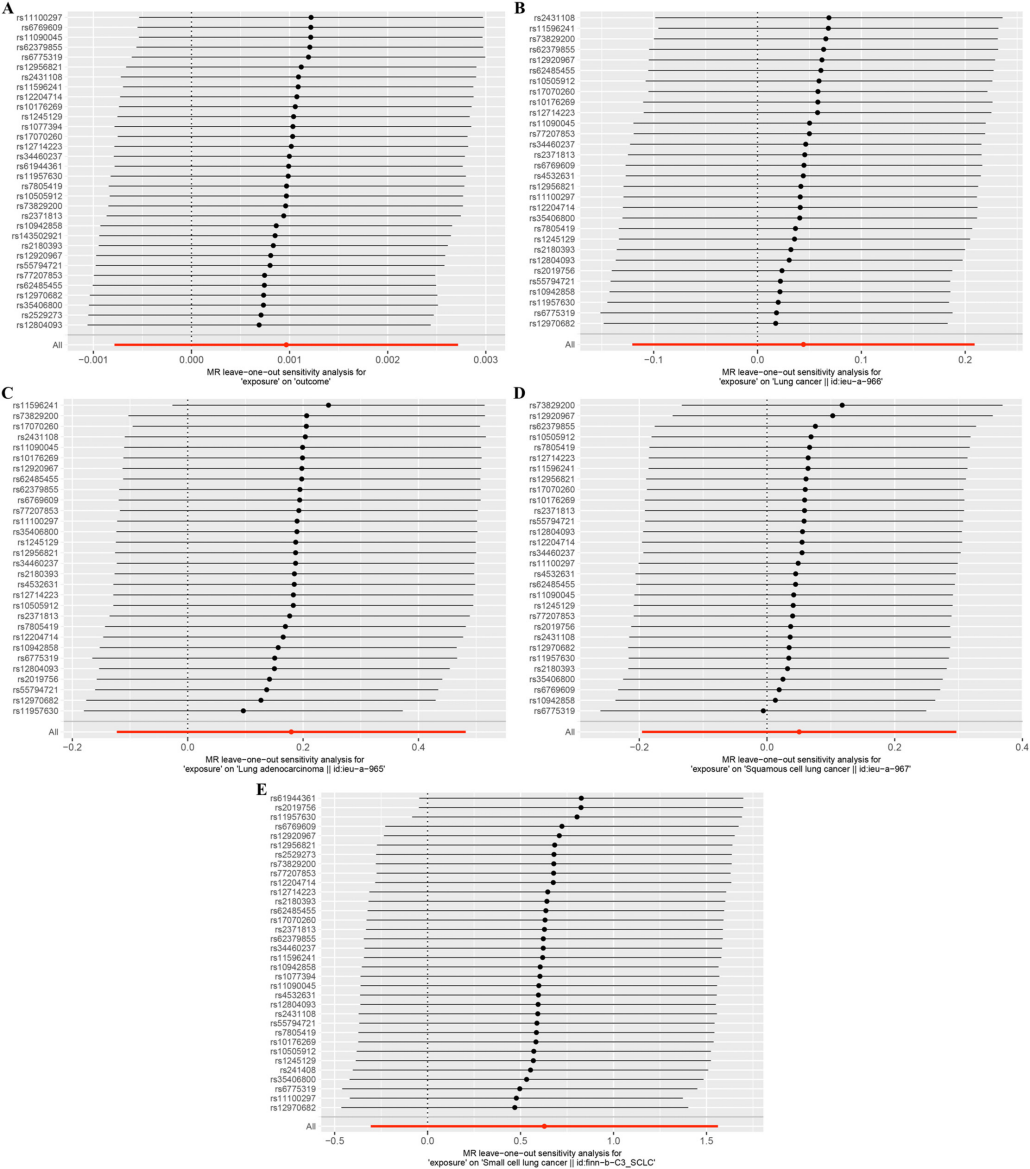
Sensitivity analysis of the association between antidepressant use and lung cancer. A. MR leave‐one‐out sensitivity analysis for “exposure” on “Outcome”; B. MR leave‐one‐out sensitivity analysis for “exposure” on “Lung cancer”; C. MR leave‐one‐out sensitivity analysis for “exposure” on “Lung adenocarcinoma”; D. MR leave‐one‐out sensitivity analysis for “exposure” on “Squamous cell lung cancer”; E. MR leave‐one‐out sensitivity analysis for “exposure” on “Small cell lung cancer”.

Sensitivity tests were performed to evaluate the reliability of the MR findings. The Cochran's Q test indicated potential heterogeneity when analyzing antidepressant use and LUAD (*p* = 0.040); however, the heterogeneity was controlled in the random‐effects IVW model, and no other analyses showed significant heterogeneity (*p* > 0.05 across other comparisons). To evaluate pleiotropy, MR‐PRESSO identified no outliers, and MR‐Egger regression indicated no evidence of significant horizontal pleiotropy. Additionally, a leave‐one‐out analysis verified the robustness of the MR findings, as no single SNP substantially influenced the overall effect estimates. These findings are summarized in Table 2, and Figure 3 provides a visual summary of the leave‐one‐out sensitivity analysis, highlighting the validity of our conclusions.

**TABLE 2:** Sensitivity analysis results.

Outcome	Cochran Q test		MR‐Egger		MR‐PRESSO
	Q value	*p*	Intercept	*p*	*p*
ieu‐b‐4955	31.913	0.421	−5.191E05	0.835	0.288
ieu‐a‐966	30.237	0.402	0.038	0.088	0.603
ieu‐a‐965	43.575	0.040	0.060	0.131	0.254
ieu‐a‐967	25.244	0.666	0.023	0.487	0.670
finn‐b‐C3_SCLC	38.907	0.221	0.062	0.601	0.197

## 4 Discussion

Our findings indicate no causal relationship between antidepressant use and lung cancer risk or its histological subtypes. This suggests that previous observational associations might be attributed to confounding factors such as smoking or socioeconomic status, rather than a direct pharmacological effect of antidepressants.

To date, there is no consistent evidence establishing a link between antidepressants and lung cancer. Many clinical trials have found the opposite. Many clinical trials have reported no increased risk. In a nested case–control study using a large, representative UK population database, researchers examined the associations between lung cancer and the use of SSRIs, TCAs, and SNRIs. They found that both SSRIs (OR = 1.27, 95% CI = 1.16–1.38) and TCAs (OR = 1.45, 95% CI = 1.31–1.60) were linked to a high lung cancer risk [12]. However, another case–control study using the Taiwan National Health Insurance Research Database found no association between any category or dosage of antidepressants and lung cancer incidence [36]. Notably, these studies lack critical information on potential confounders, such as smoking, alcohol consumption, and family history of lung cancer, factors likely to influence the incidence of both depression and lung cancer in the studied populations. A meta‐analysis of 11 observational studies reported a modest association between antidepressant use and lung cancer risk, indicating an 11% increased risk (RR = 1.11; 95% CI = 1.02–1.20; *n* = 6), though no effect on overall survival (RR = 1.04; 95% CI = 0.75–1.45; *n* = 4) [21]. Smoking is an important risk factor for lung cancer, particularly for squamous cell carcinoma. A recent large retrospective cohort study reanalyzed the relationships between antidepressant use and lung cancer in both smokers and non‐smokers to account for the confounding effect of smoking. The study found that long‐term antidepressant use was linked to a low lung cancer risk in both groups (OR = 0.61; 95% CI: 0.46–0.80 for smokers; OR = 0.75; 95% CI: 0.65–0.86 for non‐smokers) [22]. Although the study was stratified by sex, age, race, and comorbidities, it did not account for other potential confounders (e.g., BMI and family history) nor did it analyze lung cancer risk by specific histological types.

Our study offers several advantages. First, while RCTs are the gold standard for assessing causality, they can be costly and time‐consuming. The MR design enables us to approximate the rigor of an RCT within an observational framework in a more cost‐effective and ethical manner, minimizing confounding bias by using randomly assigned SNPs established at conception. Second, unlike other observational methods, MR is not affected by reverse causation. Third, the two‐sample MR approach allows for a relatively large sample size, enhancing the robustness of our findings. Given the high prevalence of both depression and lung cancer in the general population, establishing evidence for a causal link between antidepressant use and lung cancer risk could significantly inform public health strategies focused on early prevention and timely intervention. Finally, our study may provide additional information for clinical decision making, and the results of the study suggest that health professionals may take less account of lung cancer risk when prescribing antidepressant classes to patients.

However, this study also has limitations. First, the generalizability of our findings to diverse populations remains uncertain, as all the GWAS data are derived from subjects of European ancestry and may not extend to other ethnic groups. Future studies should include a wider range of population groups to further improve the applicability and relevance of the findings across different populations. Second, this study did not provide a detailed analysis of potential sources of bias, issues related to pleiotropy, and the selection of genetic markers used; future elaboration of these issues would further enhance the reliability of the study. Third, we did not find GWAS data related to antidepressant class (SSRIs, SNRIs, TCAs, etc.) or dosage, which prevented us from further refinement. Finally, the current GWAS database lacks detailed summary statistics for different types and durations of antidepressant use, preventing further investigation into potential associations between specific antidepressant classes and lung cancer risk.

## 5 Conclusions

This is the first MR analysis to comprehensively assess the causal relationship between antidepressant use and lung cancer risk. Our findings suggest that increased lung cancer screening in individuals with a genetic predisposition toward antidepressant use may be unnecessary. The potential impact of antidepressants on lung cancer prognosis, however, warrants further investigation.

## Author Contributions

**Chunli Yang:** conceptualization, data curation, and writing – original draft. **Wenlin Xu:** project administration, writing – review and editing. All authors approved the manuscript and this submission.

## Ethics Statement

This study analyzed publicly available summary statistics; therefore, ethical approval was not required.

## Conflicts of Interest

The authors declare no conflicts of interest.

## Supporting information


**Table S1.** Supplementary information.

## Data Availability

Outcome data is available on the UK Biobank (https://www.ukbiobank.ac.uk/), exposure data is available on the FinnGen database (https://www.finngen.fi/en/access_results), and GWAS summary data is available on the IEU OpenGWAS database (https://gwas.mrcieu.ac.uk/).
